# Integration of a Digital Health Intervention Into Immunization Clinic Workflows in Kenya: Qualitative, Realist Evaluation of Technology Usability

**DOI:** 10.2196/39775

**Published:** 2023-03-14

**Authors:** Samantha B Dolan, Rachel Wittenauer, Jessica C Shearer, Anne Njoroge, Penina Onyango, George Owiso, William B Lober, Shan Liu, Nancy Puttkammer, Peter Rabinowitz

**Affiliations:** 1 International Training and Education Center for Health University of Washington Seattle, WA United States; 2 Department of Global Health University of Washington Seattle, WA United States; 3 Bill and Melinda Gates Foundation Seattle, WA United States; 4 PATH Seattle, WA United States; 5 County Department of Health Siaya Kenya; 6 International Training and Education Center for Health University of Washington Nairobi Kenya; 7 Biobehavioral Nursing and Health Informatics University of Washington Seattle, WA United States; 8 Department of Industrial and Systems Engineering University of Washington Seattle, WA United States

**Keywords:** immunizations, electronic immunization registry, workflow, usability, realist research

## Abstract

**Background:**

In an effort to increase vaccination coverage in low-resource settings, digital tools have been introduced to better track immunization records, improve data management practices, and provide improved access to vaccination coverage data for decision-making. Despite the potential of these electronic systems to improve the provision of health services, few digital health interventions have been institutionalized at scale in low- and middle-income countries.

**Objective:**

In this paper, we aimed to describe how health care workers in Kenya had integrated an electronic immunization registry into their immunization clinic workflows and to use these findings to inform the development of a refined program theory on the registry’s usability.

**Methods:**

Informed by realist methodology, we developed a program theory to explain usability of the electronic immunization registry. We designed a qualitative study based on our theory to describe the barriers and facilitators influencing data entry and use. Qualitative data were collected through semistructured interviews with users and workflow observations of immunization clinic sessions. Our findings were summarized by context-mechanism-outcome relationships formed after analyzing our key themes across interviews and workflow observations. Using these relationships, we were able to identify common rules for future implementers.

**Results:**

Across the 12 facilities included in our study, 19 health care workers were interviewed, and 58 workflow sessions were observed. The common rules developed from our qualitative findings are as follows: rule 1—ensure that the users complete training to build familiarity with the system, understand the value of the system and data, and know where to find support; rule 2—confirm that the system captures all data needed for users to provide routine health care services and is easy to navigate; rule 3—identify work-arounds for poor network, system performance, and too few staff or resources; and rule 4—make users aware of expected changes to their workflow, and how these changes might differ over time and by facility size or number of patients. Upon study completion, we revised the program theory to reflect the importance of the goals and workflows of electronic immunization registries aligning with reality.

**Conclusions:**

We created a deeper understanding of the underlying mechanisms for usability of the registry. We found that the electronic immunization registry had high acceptability among users; however, there were numerous barriers to using the system, even under ideal conditions, causing a misalignment between the system and the reality of the users’ workflows and their environment. Human-centered design and human-factors methods can assist during pilot stages to better align systems with users’ needs and again after scale-up to ensure that interventions are suitable for all user settings.

## Introduction

Before the COVID-19 pandemic, approximately 25% of the children in Africa remained underimmunized against vaccine preventable diseases [1]. Vaccinations have shown to be one of the most effective public health interventions for reducing the burden of infectious diseases [2,3]. In an effort to increase vaccination coverage in low-resource settings, technological tools have been introduced to better track un- or underimmunized children, communicate vaccination appointment reminders, improve data management practices, and provide improved access to immunization coverage data for program managers and decision makers [4]. There has been an increased uptake of new data collection, management, and communication systems in low-resource health care settings because mobile phones, tablets, and laptops have become cheaper and more accessible [5]. The potential of these interventions to improve health systems was recognized by the World Health Assembly in 2018, and in 2019, the World Health Organization provided recommendations on the use of these systems for improving health outcomes based on available evidence [6,7]. Despite this global call for use of electronic systems to improve provision of immunization services, few digital health interventions (DHIs) have been institutionalized at scale in low- and middle-income countries [4,8,9].

DHI projects often fail to be effectively adopted by users or to demonstrate their potential value owing to the poor understanding of users’ needs and the implementation context [10]. This poor fit between DHI and implementation settings can be overcome through the use of human-centered design (HCD), human factors and ergonomics, and implementation science approaches [11]. These approaches have become increasingly popular for international development and social innovation projects [12]. However, HCD approaches are often only used in the initial phases of designing and deploying a DHI; implementation science research approaches are needed to supplement this methodology to study whether these interventions are effective in practice after deployment. “HCD and implementation science share the common goal of improving the use of innovative and effective practices in real-world contexts” [13]. Traditional public health research and evaluation methodology focuses on hypothesis-driven questions and methods, whereas design-thinking research accommodates iteration, prototyping, and ambiguity [14].

We aimed to identify how well health care workers (HCWs) in Kenya had integrated an electronic immunization registry (EIR) into their immunization clinic workflows using qualitative HCD and implementation science research methodologies. In particular, we focused on understanding EIR usability, including the design of the system as well as the capacity of HCWs to use data in the EIR for identifying un- and underimmunized children. We designed a realist evaluation within a mixed methods workflow modification project to describe the barriers and facilitators influencing data entry and use after EIR introduction, specifically focusing on EIR usability and acceptability. The overarching goal of the project was to assess and redesign immunization session workflows when the EIR was used at the point of care (POC) to increase user acceptability and efficiency. In this manuscript, we describe only the baseline qualitative observations, whereas the baseline quantitative and modified workflow observations are presented in separate papers [15,16].

Informed by existing theories of health-technology adoption, we developed an initial program theory and then refined the theory using realist research to more closely reflect the context in which the EIR was deployed and provide a more clear conception of the linkage between the technology and users’ experiences. Realist research aims to explain “how interventions work, for what populations, and under what circumstances” [17,18]. Realist program theories characterize configurations of contexts, mechanisms, and outcomes to construct hypotheses about social phenomena [19,20]. We perceived that this methodology would provide a more dynamic and meaningful interpretation of our qualitative data. The objectives of our study were as follows:

1. Understand EIR acceptability and users’ perceptions of facilitators of and barriers to EIR use.
2. Develop and refine a program theory on EIR usability using findings from our study.
3. Create rules for EIR design and deployment for future DHI implementers using context-mechanism-outcome configurations.

The development of the program theory allowed us to build off of the findings of other empirical studies and then use our findings to revise the theory to better outline the underlying mechanisms and contextual factors affecting usability.

## Methods

Methods are reported according to the Consolidated Criteria for Reporting Qualitative Research) checklist and the Realist and Meta-narrative Evidence Syntheses: Evolving Standards II reporting standards for realist evaluations [20,21].

### Study Setting

Siaya County is located in Western Kenya along Lake Victoria and has a population of 993,183 people as of 2019, with most living in a rural environment, and 41% aged <15 years [22]. According to the 2014 Demographic and Health Survey, 78% of the children in Siaya County were fully vaccinated [23]. At the time of this study, multiple DHI projects were being deployed across the county, and some HCWs included in our study were involved in other projects.

### Intervention Description

The EIR was introduced to all immunizing facilities in Siaya County starting in 2018. A tablet-based EIR application was designed and developed using open-source software (OpenSRP-OpenMRS) adapted for the Kenyan health care setting to reflect the country’s immunization program schedule, closely reflecting the standard paper-based reporting forms used by HCWs during immunization sessions, but with clinical decision support features to guide clinicians. It was designed as a tablet-based POC system with web-based or offline functionality connected to a central data repository. Information on a child registered in the EIR could be viewed and edited from any tablet when the system was web-based. The EIR collected child demographic and contact information along with their vaccination records, receipt of vitamin A and use of insecticide-treated nets, as well as height and weight information for growth monitoring. The EIR was not interoperable with the national DHIS2 routine health information system at the time of this study.

The EIR was rolled out to all 161 immunizing facilities throughout the county through several phases of training. It was first piloted in Gem subcounty in early 2018 at 10 health facilities. This was followed by introduction to an additional 10 facilities in the same subcounty in late 2018. The software was tested and upgraded before being scaled-up in early 2019 to the remaining 141 immunizing health facilities. A cascade-training approach was used. The Ministry of Health (MOH) and the International Training and Education Center for Health (I-TECH) Kenya staff members served as the master trainers who trained 1 to 2 staff members from each facility. Each facility received 1 tablet immediately after training. A WhatsApp group was established and maintained by the MOH and I-TECH to create a peer-support network for users and allow for remote troubleshooting.

It should be noted that before the data collection for this study, the EIR software was upgraded, which anecdotally solved some of the known software bugs but slowed the system’s performance and caused it to sometimes shut down unexpectedly.

### Implementation and Data Entry

At each immunizing facility, upon completion of training and receipt of a tablet, HCWs were expected to immediately begin using the EIR, first by entering information from the facility’s paper-based immunization registry for the preceding 12 months and then by prospectively entering vaccination data for every child seen for services thereafter. Following the MOH guidance, HCWs using the EIR completed dual data entry, inputting patient information into paper-based tools and the EIR either concurrently at the POC or retrospectively to simultaneously maintain records, at the end of a clinic session. All facilities were required to maintain up-to-date paper records throughout the study period.

### Research Design

For realist evaluation in our research, we first developed an initial program theory on EIR usability to explain how an EIR could theoretically improve the use of data for decision-making. We defined usability as the technology’s “quality of use,” considered as “the degree to which a product or system can be used by specific users to meet their needs to achieve specific goals with effectiveness, efficiency, freedom from risk, and satisfaction in specific contexts of use” [24]. We were interested in understanding the relationship between data accessibility and use, rather than the EIR’s impact on health-related outcomes.

**Figure 1:**
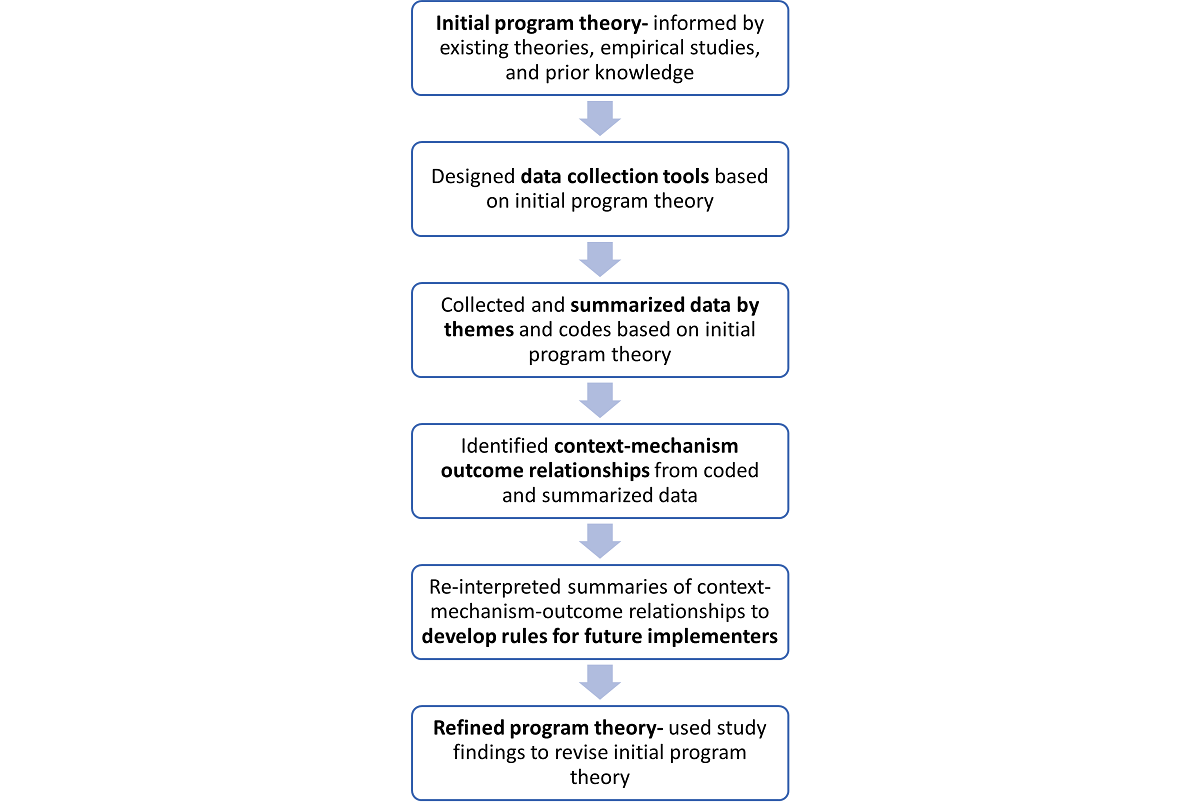
Overview of theory development and data synthesis.

We developed our initial program theory using prior knowledge of health-technology evaluations, existing theories of health-technology adoption and a targeted search of empirical studies [18] (Figure 1). We considered the Fit Between Individuals, Task, and Technology framework, which describes evaluating the fit among individual, task, and technology for improved user adoption and the Smith ergonomics balance theory of job design for stress reduction, which expands from the Fit Between Individuals, Task, and Technology to include physical environment and organizational conditions [25,26]. We used findings from empirical studies and evaluations to identify variations in the conditions to iterate on the program theory, considering how our study’s findings on EIR usability were reflected by the initial program theory and which intermediary conditions could affect usability.

### Data Collection

Facilities were purposively selected from a sampling frame that included all public facilities in Siaya County performing POC data entry and administering vaccines daily. On the basis of this list, we chose 12 facilities based on size and length of experience using the EIR and considered the accessibility of the facility for data collection. We hypothesized that smaller facilities would experience more challenges using the EIR over paper-based tools than larger facilities and that large facilities would require additional resources to sustain the use of EIR. Facility size was determined by the number of children aged <1 year in the facility’s catchment area, using the categories of small, medium, and large, based on the 33rd and 66th quantiles of the sampling frame. The length of experience using EIR was categorized as <3 months and ³3 months. One to 2 HCWs at each facility most familiar with the EIR were selected for interviews. Usually they had been formally trained on the EIR but some learned on the job. Subcounty health records information officers (SCHRIOs) overseeing EIR deployment among the selected facilities were also interviewed on their perceptions of facilitators and barriers among the staff members whom they supervised. We determined that if data saturation was not reached by the time data collection was completed at all 12 facilities, we would institute a stopping criterion of 2 facilities (per category) until we reach saturation based on the types of challenges observed during workflow observations [27].

Qualitative data were collected through semistructured interviews with users and direct workflow observations of immunization clinic sessions; data collection tools were driven by the initial program theory. Direct observation is a standard method in human-factors research and considered a useful technique when studying how technology changes user workflows and tasks [28-30]. In July 2019, two researchers (SD and RW) with previous experience in collecting qualitative data trained 4 local data collectors on how to conduct semistructured interviews and workflow observations. Data collectors had previous experience in conducting surveys and had no prior relationships with the interviewees. Interviews were conducted in-person after observation of an immunization session. The facility visits were facilitated by the SCHRIOs, who introduced the data collectors to the facility staff members and were sometimes present at the facility during the interviews.

The standardized semistructured interviews included open-ended response questions and questions with Likert scale responses (scale 1-5) to indicate level of agreement on EIR usability based on the expanded usability heuristics by Nielsen (details on data collection tools are included in the Multimedia Appendix 1) [31]. Responses were marked on paper forms and data collectors took notes on open-ended interview questions and workflow observations in real time. Interview responses were recorded on paper and later were input into a spreadsheet for cleaning and analysis.

To document user workflows, data collectors used a standardized tool to observe and document the workflow of HCWs providing services to children seen in the immunization clinic for vaccinations or growth monitoring. Data collectors were instructed to stand in the immunization room and observe an entire session, usually conducted in the morning, until at least 5 children had been observed. Each facility’s workflow was documented, including the sequence of activities, characteristics of the child being seen, and the number of staff working during the immunization session. Interruptions and other environmental observations were noted. Data were collected on paper forms and later entered into a web-based Google Form.

### Data Analysis

We calculated the frequencies of facility and interviewee characteristics. For the workflow observations and Likert scale responses, we used descriptive statistics to summarize the number of workflows by type, order of activities, and frequency of activities. For the qualitative data, the researchers wrote memos on key usability-observations for each interview after data collection. The memos along with the initial program theory were used to develop themes and codes to summarize the open-ended questions. A code list was created and then piloted using 3 interviews. For every response, the coder had to choose the most appropriate theme and at least one code; some codes were further classified into subcodes. SD and RW used ATLAS.ti to independently code each interview, then reviewed discrepancies, and came to a consensus. Codes were updated after the initial round of review, however, only for those concerning usability, because more specificity was needed (see Multimedia Appendices 2 and 3 for codes and definitions). All data were managed and summarized in Microsoft Excel.

### Synthesis of Rules

To better understand EIR usability, we summarized our findings by context-mechanism-outcome relationships formed after analyzing our key themes across interviews and facility memos. Context was considered as the conditions in a given setting that could influence the mechanism-outcome relationships, either positively or negatively. We considered mechanisms to indicate underlying processes needed to generate the outcomes. As indicated by our study objective, the outcomes of interest in this study were users’ acceptance of the EIR when used at the POC alongside paper-based tools and usability of the EIR data.

We summarized the coded data by the context-mechanism-outcome configurations and then reread and reinterpreted the data looking for patterns, common themes, and negative evidence to identify emergent rules that could be used by future implementers in low-resources settings to successfully introduce a DHI. Proposed rules were discussed with and agreed upon by other research team members. We continued to iterate upon the rules, guided by the summaries, until the rules were able to appropriately capture our findings. These rules were reviewed by RW to ensure that they aligned with the study findings and then used them to describe the final results and to describe how they related to the refined program theory. Respondents did not provide feedback on findings.

### Ethics Approval

This study was determined as non–human subjects research by University of Washington Institutional Review Board (STUDY00006256), given that no biological specimens were collected, and received human subjects ethics approval from Amref Health Africa-Kenya (ESRC P587-2019) and the US Centers for Disease Control and Prevention (GCH HSR #: 2018-293) because it was considered part of routine program evaluation. The interview team received consent from all participants to complete the semistructured interviews. SD, RW, and JS led the design, implementation, and interpretation of findings for this study. SD advised on the design and implementation of the EIR.

## Results

### Facility and Health Care Worker Characteristics

Of the 12 facilities purposively sampled, 6 (50%) facilities had <3 months of experience in using the EIR and 4 (33%) facilities fell into each facility size category (Table 1). A total of 10 (83%) facilities were public, and the remaining 2 (17%) were faith-based. Moreover, 10 (83%) facilities administered vaccinations daily. All 12 (100%) facilities had electricity supply; however, only 2 (17%) had a backup power supply. Of the 19 interviewees, 14 (74%) had been working at the facility for 1 to 5 years, 10 (53%) had ≥3 months of experience using the system, and 12 (63%) were nurses.

**Table 1:** Facility and health care worker characteristics.

Characteristics				Values
**Facility characteristics (n=12)**				
	**Length of time using the EIR^a^, n (%)**			
		<3 months	6 (50)	
		≥3 months	6 (50)	
	**Facility type, n (%)**			
		Dispensary	4 (33)	
		Health center	6 (50)	
		County referral hospital	2 (17)	
	**Facility size, n (%)**			
		Small	4 (33)	
		Medium	4 (33)	
		Large	4 (33)	
	**Facility ownership, n (%)**			
		Faith-based	2 (17)	
		Public	10 (83)	
	**Vaccines administered daily, n (%)**			
		Yes	10 (83)	
	**Facility has electricity, n (%)**			
		Yes	12 (100)	
	**Facility has backup power, n (%)**			
		Yes	2 (17)	
	**Staffing, mean (SD)**			
		Average number of nurses stationed in the immunization clinic	2.6 (1.8)	
**Health care worker characteristics (n=19)**				
	**Years working at facility, n (%)**			
		<1 year	1 (5)	
		1-5 years	14 (74)	
		6-10 years	1 (5)	
		>10 years	2 (11)	
		Missing	1 (5)	
	**Time spent using EIR, n (%)**			
		1-3 months	8 (42)	
		>3 months	10 (53)	
		Missing	1 (5)	
	**Staff cadre, n (%)**			
		Nurse	12 (63)	
		Nurse in charge	3 (16)	
		Laboratory technician	1 (5)	
		Missing	3 (16)	

^a^EIR: electronic immunization registry.

### Workflow Summaries

There were no workflow observations documented at 1 facility because there were no children presenting for services. Of the 58 workflow observations completed at 11 facilities, there were 42 (72%) in which the HCW alternated between inputting information into the EIR and the paper-based tools for each activity at the POC, whereas for 5 (9%) observations, the HCW only used paper-based tools and later entered data into the EIR, and for 11 (19%) observations, only paper-based tools were used. When the EIR was not used at the POC, it was because of the issues with system performance.

**Figure 2:**
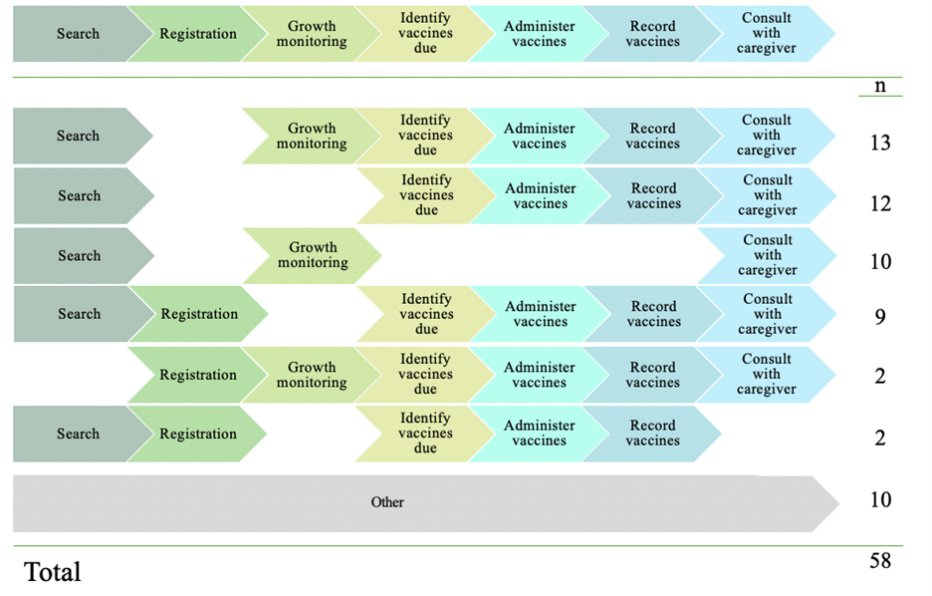
Frequency of workflow types by order of activities.

Of the 58 workflows observed, no vaccines were administered during 12 (21%) of the observations (Figure 2). Generally, the workflows included a similar order of activities; the most common (13/58, 22%) workflow was observed among children returning to the facility, having already been registered in the EIR, where the HCW searched for their record, conducted growth monitoring, identified vaccines due, administered and recorded the vaccines, and then provided a consultation with the caregiver. Workflows varied by whether the child needed to be registered, needed growth monitoring, was due for vaccine administration, and whether a consultation with the caregiver was provided.

Users’ agreement with the ease of use of the EIR at the POC was assessed using standard heuristics as presented in Table 2. Generally, there was a high level of agreement (≥75%) with EIR usability; however, users disagreed on the EIR being well integrated into their workflow, with 32% (6/19) disagreeing on having a good workflow when completing dual data entry and 42% (8/19) disagreeing on having enough staff to adequately use the EIR in an immunization clinic.

**Table 2:** User agreement on usability of an electronic immunization registry at the point of care.

Usability category and usability statements			All facilities						
			Values, median (range)		Disagree or strongly disagree, n (%)		Neither agree nor disagree, n (%)		Agree or strongly agree, n (%)
**Visibility of system status**									
	1. “You feel the system provides enough feedback/messages to you as it processes information, you understand what the system is doing.”	4 (2-5)		1 (5)		0 (0)		18 (95)	
**Match between system and real world**									
	2. “You feel that the EIR^a^ captures the correct information during a nutrition and immunization session.”	4.5 (2-5)		3 (16)		0 (0)		16 (84)	
**User control and error prevention**									
	3. “You feel you have the ability to undo and redo actions when entering child information.”	4 (1-5)		1 (5)		0 (0)		18 (95)	
	4. “If you make an error when using the EIR, you feel it is easy to correct the mistake and that the system helps you prevent making mistakes.”	4 (1-5)		2 (11)		0 (0)		17 (89)	
	5. “It is easy to move from one screen page/menu to the next.”	5 (2-5)		1 (5)		0 (0)		18 (95)	
	6. “You can easily update/edit all the child registration details.”	5 (2-5)		1 (5)		1 (5)		17 (89)	
**Esthetic and minimalist design**									
	7. “You feel there is not too much information in the EIR, and that all of the information is needed.”	4 (1-5)		2 (11)		1 (5)		16 (84)	
**Flexibility and efficiency of use**									
	8. “You feel the system is flexible enough to allow you to complete frequent actions easily.”	4 (2-5)		1 (5)		0 (0)		18 (95)	
	9. “I find the EIR easy to use.”	5 (4-5)		0 (0)		0 (0)		19 (100)	
**Help and documentation**									
	10. “You feel it is easy to find help when you need it.”	4 (2-5)		3 (16)		1 (5)		15 (79)	
**Help users recognize, diagnose, and recover from errors**									
	11. “You feel that the error messages the EIR generates help to indicate a problem and suggest how to solve it.”	4 (2-5)		2 (11)		3 (16)		14 (74)	
**Integration into real-time workflow**									
	12. “The EIR provides the information I need to easily vaccinate children.”	5 (4-5)		0 (0)		0 (0)		19 (100)	
	13. “I have enough time to vaccinate all patients attending an immunization clinic.”	4 (2-5)		1 (5)		0 (0)		18 (95)	
	14. “The clinic workflow is good when using the EIR and paper tools at the point-of-care.”	4 (2-5)		6 (32)		1 (5)		12 (63)	
	15. “We have enough tablets for our clinic to use the EIR.”	4 (1-5)		3 (16)		0 (0)		16 (84)	
	16. “We have enough staff to adequately use the EIR during our immunization clinic.”	3.5 (1-5)		8 (42)		1 (5)		10 (53)	
	17. “The system was functioning well most of the time when you needed to use it.”	4 (1-5)		4 (21)		1 (5)		14 (74)	
	18. “System downtime was minimal.”	4 (2-5)		1 (5)		1 (5)		17 (89)	
	19. “I trust that the data in the EIR is stored securely and will not be lost.”	4 (1-5)		1 (5)		0 (0)		18 (95)	
	20. “I trust the data in the EIR are of good quality.”	5 (2-5)		1 (5)		1 (5)		17 (89)	
**Satisfaction**									
	21. “I would recommend the system for use by other users/health facilities”	5 (4-5)		0 (0)		0 (0)		19 (100)	
	22. “Overall, I am satisfied with the EIR.”	4 (4-5)		0 (0)		0 (0)		19 (100)	
	23. “The EIR improves the quality of patient care.”	4 (4-5)		0 (0)		0 (0)		19 (100)	
	24. “I feel I received adequate training on how to use the EIR appropriately for my clinic.”	4 (2-5)		4 (21)		1 (5)		14 (74)	
	25. “I know where to find the EIR user guides and help functions.”	4 (1-5)		4 (21)		0 (0)		15 (79)	
	26. “I feel I receive adequate supervisory support for using the EIR in my clinic.”	4 (1-5)		3 (16)		1 (5)		15 (79)	

^a^EIR: electronic immunization registry.

### Program Theory

Our initial program theory illustrated EIR usability as a cyclical relationship among data demand, data capture and accessibility, and data used for decision-making, which covers activities such as identifying clients due for vaccination, scheduling future visits, and retrieving clients’ contact information (Multimedia Appendix 4). Poor data demand, accessibility, and use have been observed because of the weaknesses at individual, organizational, and infrastructural levels, leading EIRs to serve little utility for decision-making [6]. We included these levels as components of the enabling environment for our intervention theory (Multimedia Appendix 4). At the individual level, we considered how EIR usability was dependent on a user’s capacity and personal motivation. As described in the self-determination theory, feelings of competence and a sense of autonomy can enhance intrinsic motivation for a worker to perform a task [32]. EIR users are likely to internalize their use and demand for data as they find more interest, meaningfulness, and satisfaction with activities. Individuals who perceive their work as meaningful are more committed to their organization, more engaged, and more productive [33]. At the organizational level, we considered the impact a program’s culture, policies, and hierarchy may have on EIR use; if culture and policies do not accommodate new technology, usage is likely to remain low. For infrastructure, including available resources, we considered how EIRs require technical infrastructure that is routinely maintained and upgraded, as well as sufficient staffing levels and program funding to support users and maintain equipment. The use of an EIR alone will not improve immunization coverage, other environmental enablers mediate the impact of EIR usability on coverage.

**Figure 3:**
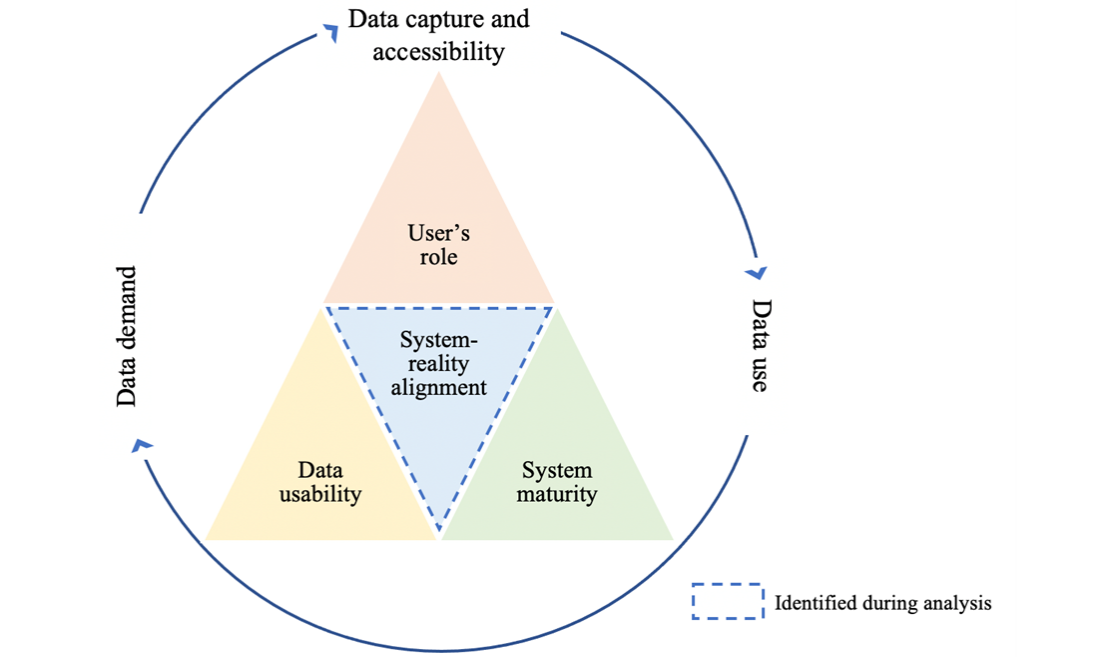
Refined Program Theory of EIR-Usability for Improving Immunization Coverage. System maturity: “the extent to which digital technologies are used as enablers to deliver a high-quality health service”, and dictates system fidelity, the accessibility and usability of the EIR data, and how data are used for health care decision-making. Data usability: “degree to which data are of sufficient quality (accuracy), completeness, timeliness to allow for effective decision making.” User’s role: specific job tasks, responsibilities, and expectations in a given position and setting. System-reality alignment: the congruence between the system, the user’s needs, and real-time workflow. Enabling environmental factors (not pictured): factors influencing EIR usability include the existing infrastructure and resources, such as the technical infrastructure, system maintenance and upgrades, staffing, and program funding, as well as an individual’s characteristics, such as competency, satisfaction, and motivation, all of which can be further influenced by organizational structure, which would include the program’s culture, policies and guidance, and organizational hierarchy and responsibilities. EIR: electronic immunization registry.

For our refined program theory we expanded the initial theory and added the intermediary conditions of system maturity, user’s role, and data usability based on empirical study findings, including our own (Figure 3). System maturity refers to “the extent to which digital technologies are used as enablers to deliver a high-quality health service” and dictates system fidelity, the accessibility and usability of the EIR data, and how data are used for health care decision-making [34]. Data usability refers to the “degree to which data are of sufficient quality (accuracy), completeness, timeliness to allow for effective decision making” [35]. Existing literature suggests that because HCWs use data more and use the full range of EIR functionality, they can improve the quality of data by identifying inconsistencies and may start to demand more high-quality data, which improves their trust in the data, and therefore reinforces data use [36,37]. The role of the user refers to their specific job tasks, responsibilities, and expectations in a given position and setting; for instance, if the user was required to use the EIR, but the EIR did not provide information needed to perform a job task, this would be poor use of the EIR, given the user’s role. Note that the conditions are not mutually exclusive and there is much overlap between them when framing their effect on EIR usability.

On the basis of our study findings, described in subsequent sections, we revised the program theory to better reflect the importance of information systems aligning with reality and included this as an additional intermediary condition. Alignment refers to the congruence between the system’s design and functionality, the needs of user’s information, and real-time workflow. This definition covers whether the system is simple enough to navigate at the POC, collects all information routinely used, and allows for data to be easily accessible to perform routine tasks. This helped to clearly emphasize the importance of the EIR accommodating the other intermediaries to support the cycle of data demand, data capture and accessibility, and data use. We carried through the inclusion of the enabling environment from the initial program theory because we found that factors such as high user workloads, untrained staff members, or poor internet connectivity could greatly influence EIR usability. Using the refined theory, we captured how EIRs can improve the capacity of users to effectively serve patients; however, poor alignment between the system and reality could decrease the system’s usability and ultimately effectiveness.

### Context-Mechanism-Outcome Configurations and Rules

**Figure 4:**
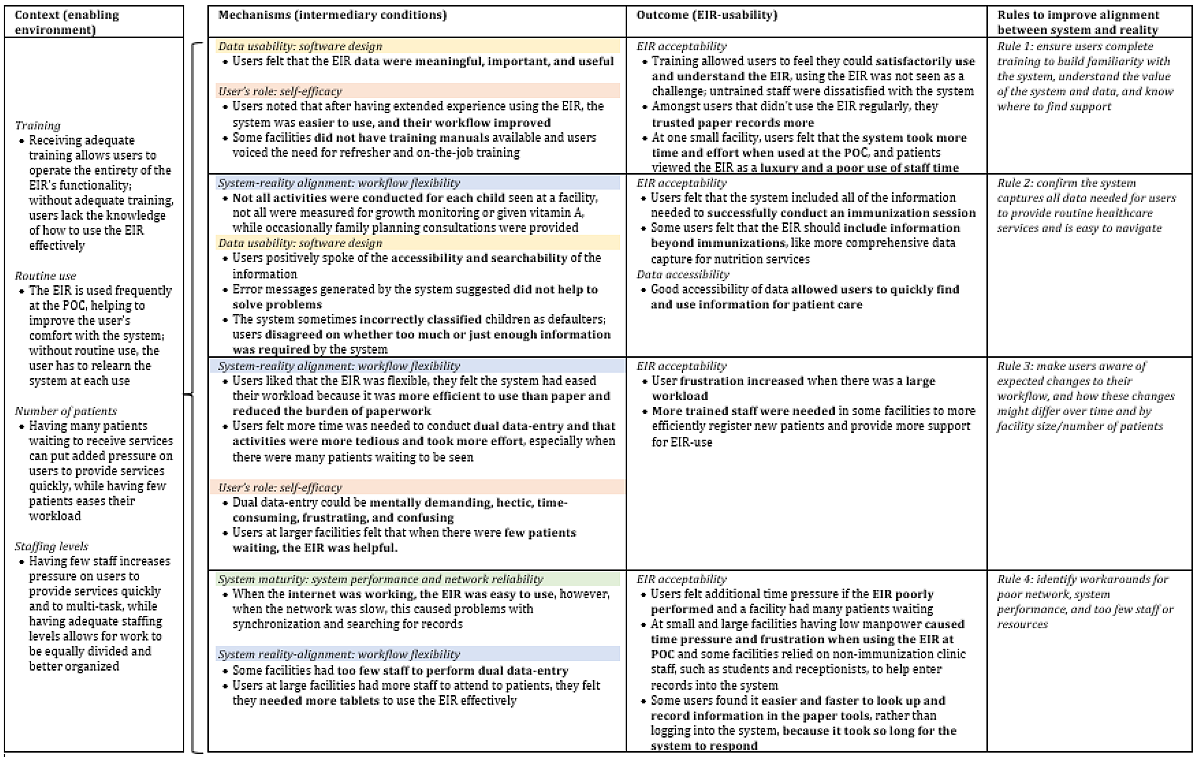
Linkages between electronic immunization registry (EIR) usability rules and the refined program theory. POC: point of care.

Our analysis identified workflow flexibility, software design, system performance and network reliability, and self-efficacy as mechanisms that reflected the intermediary conditions of our refined program theory. Context characteristics were related to the enabling environmental factors of our theory and included, staffing levels, number of patients, training, and routine use of the EIR. Our outcomes of interest, EIR acceptability and data accessibility, were selected a priori and reflected by the existing cyclical EIR usability relationship; however, we did not find emergence of any new outcomes during analysis. The addition of system reality alignment to the refined program theory was considered the key condition needed to tie together the context-mechanism-outcome configurations and was used to guide the crafting of rules for future DHI implementers (Figure 4).

### EIR Usability Rules

#### Rule 1: Ensure Users Complete Training to Build Familiarity With the System, Understand the Value of the System and Data, and Know Where to Find Support

Users’ perceptions and experience with training influenced their feelings about EIR usability and alignment between the system, their workflows, and capacity. For instance, untrained staff were often dissatisfied with the system, indicating a lack of self-efficacy. Training allowed users to feel that they could satisfactorily use and understand the EIR. Users felt confident after completing training, and using the EIR was not seen as a challenge. One user only trusted the data they personally had entered into the system because the other HCWs in their facility had not been trained and therefore could not be trusted for entering the data correctly. Working with untrained staff members caused dissatisfaction with the system among some users. Training time was believed to be too limited and some users felt it was hard to retain all the information provided, further decreasing self-efficacy, although 74% (14/19) of the users believed that they received adequate training. However, the SCHRIOs felt that the transition to the EIR occurred too quickly and that users needed to be trained longer. We observed several staff using the EIR who had received on-the-job training (OJT), whereas other staff members who had been formally trained were working in other areas of the facility at the time of observation.

In terms of software design, the EIR data was seen as meaningful, important, and useful by users, with 89% (17/19) of users feeling that they were of good quality. The system was perceived as a safe and confidential data source; 95% (18/19) of users trusted that the data stored in the EIR were secure. They trusted the data because it could not be tampered with and liked that the system rejected incorrect information and that mistakes could be corrected later. Users felt that the EIR had a positive impact on data quality because the records were backed up. Among users who did not use the EIR regularly, trust for paper records was more, because those were seen as more reliable. At one small facility, an HCW felt that patients viewed the EIR as a luxury and a poor use of staff time.

Some facilities did not have training manuals available and users voiced the need for refresher and OJT; 21% (4/19) of users who were interviewed did not know where to find user guides. In terms of staffing, some facilities had IT staff members available to help maintain the EIR, whereas others required SCHRIOs to assist or used WhatsApp groups for assistance; 79% (15/19) of users agreed that it was easy for them to find help; however, receiving assistance immediately was not easy for all facilities and not all facilities received supervision visits. In addition, SCHRIOs believed that there was a gap in the support structure, further supported by our observation that 16% (3/19) of the users believed that they did not receive adequate supervisory support.

#### Rule 2: Confirm That the System Captures All Data Needed for Users to Provide Routine Health Care Services and Is Easy to Navigate

All users who were satisfied with the EIR, agreed that it improved patient care, and would recommend the system to other users or health facilities (Table 2). EIR users believed that the system included all the essential information needed to successfully conduct an immunization session. However, there were important differences observed for each child seen for services, of which some were coming in for immunizations whereas others were being seen for a well-child visit; this required flexibility in user workflows and software designed to accommodate differing visit types. We observed that not all activities were conducted for each child seen at a facility; for instance, not all children were measured for growth monitoring or given vitamin A, although occasionally family planning consultations were provided to the caregiver. Some users suggested that the EIR should include information beyond immunizations, such as more comprehensive data capture for nutrition services and tetanus toxoid vaccine, which is only a recommended vaccine for adults and not included in standard childhood vaccination clinics.

There were several EIR software updates needed to make it a more user-friendly design. Users noted that the recommended childhood vaccine schedule was not presented correctly in the EIR owing to software bugs along with the need for vitamin A and insecticide-treated net schedules to be updated. The system sometimes incorrectly classified children as defaulters because of the software bugs, which caused some users to trust the paper records over the EIR. Users disagreed on whether too much or just enough information was required by the system. In addition, the name of a child’s father should have been included in the registration form, and several other fields should be made optional because caregivers cannot always provide this information at the time of registration. Some defaulters were displayed incorrectly, some users could not capture those children living outside the county, and it was sometimes difficult for users to update a child’s registration information.

Users positively spoke of the efficiency of the system for storing patient information as well as the accessibility and searchability of the information. Others noted they felt less time pressure to perform tasks when using the system because they trusted the data inputted and found the system easy to navigate, especially when it became routine and they used it every day. Some users felt the EIR was easier to use than paper tools and made their work less burdensome; all (n=19) users agreed that the system was easy to use. However, we did observe users that still relied on paper wall calendars to identify a child’s next vaccination date, rather than use the automatically generated date from the EIR. Despite the challenges, the SCHRIOs also felt the EIR was easy to use and were satisfied with the system. They observed that HCWs were not hesitant to use the EIR and were impressed by the system.

In terms of system functionality, it was mentioned that error messages could be unclear or difficult to understand, 11% (2/19) of users did not feel that the error messages generated by the system suggested how to solve problems. We observed that when the EIR was lagging, some users had difficulty understanding if all information had been saved, as the EIR did not give notification. among all users interviewed, 95% (18/19) agreed that the system provided enough feedback, 84% (16/19) agreed the EIR captured the correct information, 89% (17/19) believed errors could be prevented and information easily updated, and 95% (18/19) agreed that it is easy to navigate through the system. The clinical decision support features reminded users of which services or vaccines were due for a particular patient, in addition to quickly identifying which patients were defaulters. The EIR generated the monthly report of the total number of children vaccinated, which allowed for easy summation of data and for users to understand trends in the data. Users liked that the tablet was more portable than paper records and that the system guided the user on what to do next during a patient visit and they could easily move within the system. Users felt that updating information in the system was easy. Some users noted that using the EIR to search for children registered outside of the facility could be difficult because it required data syncing that could be slow or not possible if there was no internet connectivity but did like this system feature.

#### Rule 3: Make Users Aware of Expected Changes to Their Workflow and How These Changes Might Differ Over Time and by Facility Size or Number of Patients

We heard mixed feelings about dual data entry during a clinic session. Some users felt it could be mentally demanding, hectic, time consuming, frustrating, and confusing, subsequently hampering their self-efficacy to use the system at the POC. We observed that some users waited until an immunization session was completed before entering information into the EIR in an effort to reduce their workload. Since beginning to use the EIR, some users felt that their workload had increased, but that their duties had not changed. Several users preferred using paper records because they could more quickly record information. However, in terms of workflow flexibility, other users liked that the EIR was flexible, the system had eased their workload because it was more efficient to use than paper, it reduced the burden of paperwork because they could copy information from the EIR into the paper tools, and it helped when completing paper records. Users noted that after having extended experience using the EIR the system was easier to use and their workflow improved. Numerous users mentioned they would prefer to only use the EIR.

Users felt more time was needed to conduct dual data entry and that activities were more tedious and took more effort, especially when there were many patients waiting to be seen. Although 95% (18/19) of users interviewed agreed that they had enough time to vaccinate all patients attending an immunization clinic, only 63% (12/19) agreed that they had a good workflow when they completed dual data entry at the POC. During busy clinic sessions, some facilities would only enter information into paper records at the POC and then enter information into the EIR after the session. It was mentioned that frustration increased during these types of sessions. At small and large facilities having low staffing levels, likely alongside high patient volumes, caused time pressure and frustration when using the EIR at the POC. When the immunization clinic was busy, users at small facilities felt more pressure and stress when performing dual data entry at the POC, especially when there were staff shortages. Whereas users at larger facilities felt that when they had few patients waiting, the EIR was helpful. In addition, users voiced that having to complete retrospective data entry took a long time, especially at large facilities. Pressure to perform activities quickly was compounded when the EIR performed poorly and a facility had many patients waiting. We did observe several sessions where a patient consult was not completed, possibly due to lack of time.

#### Rule 4: Identify Work-arounds for Poor Network, System Performance, and Too Few Staff Members or Resources

Almost all staff members interviewed mentioned an external challenge they faced to successfully use the EIR. Users explained that when the internet was working, the EIR was easy to use; however, when the network was slow, this caused problems with synchronization and searching for records. Users felt additional time pressure to perform tasks when there was poor network connectivity. We observed some users multitasking while the EIR was hanging. Sometimes the system failed to respond completely, and information could not be entered or updated. However, 74% (14/19) of users agreed that the system functioned most of the time when it was needed, and 89% (17/19) said system downtime was minimal; they felt that performing dual data entry was acceptable if the EIR was working well. Using the system required more effort when there was poor network connectivity, as connectivity was needed to log in and perform advanced record searches. When the EIR suffered from poor performance which caused hanging and sometimes failure to save data or low availability of network which preventing synching and information could not be entered or updated, users felt frustrated. Some users found it easier and faster to look up and record information in the paper tools, rather than logging into the system, because it took so long for the system to respond. The SCHRIOs believed that some facilities struggled transitioning to the EIR because of network connectivity, high patient volume, and staff shortages. They felt that the facilities needed backup power, improved network connectivity, adequate staffing, more tablets for large facilities, and access to the EIR data*.*

In terms of workflow flexibility, users at all facility sizes felt that more staff members needed to be trained on the system to ensure coverage when trained staff were unavailable and for completing real-time data entry. Some facilities had too few staff members to perform dual data entry, sometimes relying on nonimmunization clinic staff, such as students and receptionists, to help enter records into the system. Half (10/19, 53%) of the users indicated having enough staff to adequately use the EIR during an immunization session. Although users at large facilities had more staff to attend to patients, they felt they needed more tablets to use the EIR effectively, this was also mentioned by 16% (3/19) of all users. It was noted that more trained staff were needed in some facilities to efficiently register new patients and provide more support for EIR use. Staff only used the EIR and paper tools concurrently when there were few clients. To accommodate low staffing levels, we observed data clerks or community health volunteers receive OJT to help complete the EIR records while the nurse would fill in the paper-based tools. One facility had developed a work-around so the reception staff entered information into the EIR; however, these staff members were not compensated for their assistance and viewed the tasks as outside their job duties.

## Discussion

### Principal Findings

Our study identified the major barriers to and facilitators of EIR usability and created a deeper understanding of the underlying mechanisms and outcomes affecting users. We found that generally the EIR was well accepted; however, users faced numerous challenges to using the system, even under ideal conditions. The EIR incorrectly displayed key fields because of software bugs, and numerous facilities could not easily access the system or sync records owing to poor system performance. In addition, the introduction of the EIR imposed new obstacles for the users, often exacerbated by contextual factors such as whether the facility had enough staff, lacked routine use of the system, had inadequate training, and the patient load. These contextual factors were incorporated to our context-mechanism-outcome relationships and were reflected by the enabling environment in our program theory.

Users tended to have greater satisfaction with the EIR when it more closely aligned to their workflow, which we have termed as system reality alignment, and vocalized their preference for removing paper-based tools and only performing paperless data entry. We confirmed that our initial program theory describing EIR usability was upheld, but our study highlighted the importance of system reality alignment as a necessary intermediary condition. This finding has been described as the “design-reality gap,” the success or failure of a DHI is dependent on the size of the gap that reflects the tension between designing systems for the present versus the future [38]. DHIs should serve as mechanisms to improve health care provision and data use; however, they need to adapt to the realities of the users and their enabling environment to become viable data management tools.

### Software Design

As identified by our need to include system reality alignment in our refined program theory, we found that DHI design should not be dictated by a specific disease or health program and that the initial design process for the EIR was inadequate. Because immunization information had been prioritized for the EIR’s design, some growth monitoring and nutritional information was not well captured despite it being part of a general well-child visit in which HCWs must provide different types of services to the children. This was partly because of the adaptation of the software from an existing implementation of the system and immunization-focused stakeholders involved with the implementation process. Although the use of “global goods” such as OpenSRP can be monetarily beneficial for low-resource settings because they are technically free to use, they still need to be evaluated and significantly updated and upgraded to fit a new setting before they can be effectively used [39]. In addition, trade-offs must be made between system flexibility with accommodating user needs and data quality, as well as building program-specific versus more comprehensive systems [40]. HCD researchers and designers are faced with problems in both understanding current practices as well as in understanding how those practices may change in the future, and their methods should reflect this tension [41]. We can echo the voices of others, calling for implementers to place greater importance on continuous system adaptation and improvement of sociotechnical systems [42-44]. As workflows will need to be adapted over time as technology, human capacity, and health care needs change, efforts for adapting workflows could be particularly helpful, along with continuing to sensitize patients on the importance and expected changes after system introduction. Future studies using the Kenya EIR could consider whether our refined program theory is upheld as a DHI matures.

### DHI and the Evaluation Life Cycle

When deploying a DHI, implementers should consider users’ workflows during both the design and deployment phases. It is important to ensure that workflows which may vary by site and user are considered during the design phase. Especially in areas with poor access to electricity supply or internet, robust evaluations of a geographical connectivity should be conducted before introduction and the DHI should be designed not on an ideal connectivity scenario but based on reality. For this study, the I-TECH team had surveyed each facility for internet access and electricity outages but had not considered collecting detailed information on the strength of the internet connection that would have been important information during the design phase to ensure that the EIR could be used without interruption in areas with low connectivity.

Implementers should rigorously pilot-test the DHI to ensure that the benefits of the system continue to outweigh any software performance issues. It would be helpful if implementation teams decided at the onset of DHI deployment what they considered adequate performance metrics before scale-up. Having a metric for determining if the DHI is performing well can help inform whether facilities are ready to move to paperless data entry. Qualitative HCD studies can assist during pilot stages by ensuring that system usability and user acceptability are captured in time to make changes to the system, whereas implementation science methods can be used after introduction and during scale-up to assess intervention effectiveness; if usability problems are identified after scale-up, HCD methods can again be used to better understand lack of DHI adoption.

Using HCD methods to understand the fit between users and technology we were able to distill our findings into 4 rules that considered the importance of a user’s workflow and the enabling environment. Since the 1980s, health technology–focused researchers have invoked sociotechnical approaches to move conceptual understanding beyond simple cause and effect relationships to explain complex relationships between computers and outcomes. However, HCD methods are not routinely used in global health as the field has traditionally focused on medical or therapeutic interventions. The utility and benefit of HCD in global health is being more broadly recognized as a component of a public health professional’s toolkit; however, as of early 2019, few studies have been published that use HCD methods [45,46].

The perceived benefit of HCD is that it provides a structured approach to “systematize innovation in public health” and that design-thinking methodologies can provide new approaches to problem-solving in complex health systems, in which more traditional methods may fail [45,47]. There has been a continued emphasis on the importance of interactions between human behavior, organizational procedures, policies, and cultures when introducing an automated system and how new technologies need to be studied within these contexts [48]. Using HCD and implementation science methods together, we were able to observe and capture these interactions through our qualitative analysis. There is symmetry in the ideal approach to designing and implementing DHI alongside HCD or implementation science methodologies; as DHI are flexible systems that need to adapt to changes over time, their deployment and maintenance should be coupled with iterative evaluations over their life span that can continue to assess why changes are needed and how best to adapt the system. We encourage future evaluators to explore these methodologies and make them part of standard DHI evaluation practices.

### Study Strengths

To the best of our knowledge, this is the first study that combines HCD and realism for understanding the integration of a DHI into user workflows. Using realist research, we were able to build from findings in the empirical literature and develop a robust program theory that explained the underlying processes that could affect EIR usability and the influence of contextual factors. Had we simply summarized our qualitative findings by themes and codes, we would have lost the opportunity to *practicalize* these findings in the form of rules for implementers. The use of HCD research techniques for our study was helpful for illuminating key pieces of the software that were not meeting user needs, whereas the workflow observations provided additional insights needed to understand the system’s effect on clinic activities. If we had relied on only HCD methodology that prioritized assessing acceptability, we would have missed some key findings emerging from the interviews that were important for understanding the mechanisms of data use and contextual factors needed for project sustainability. Potential errors and failures can be best understood by observed system operations, and these failures can only be identified when the system is operating in its real environment. Surprisingly, we found that the use of dual data entry at facilities, rather than paperless alone, provided users with a side-by-side comparison of the 2 types of systems, which we think allowed them to make more specific and clear comments about facilitators and barriers and allowed us to understand where key pieces of information or usability gaps were in the EIR compared with paper tools.

### Limitations

We faced several limitations during this study, including the limited generalizability of our findings owing to the purposive sampling approach, biases because of the Hawethorne effect in which HCWs may have changed their behavior because they were being observed, and social desirability bias from HCWs wanting to be seen as good users of the EIR. In addition, the EIR’s software was upgraded immediately before study initiation and although, both our team and the implementation team did not expect the EIR’s performance to change because of this upgrade, we observed and heard through interviews that the upgrade greatly affected system performance. Therefore, our results reflect the findings of an unreliable system and are not representative of EIR usability in a setting in which the system is working as intended.

### Conclusions

We created a deeper understanding of the underlying processes influencing EIR usability through our refined program theory and rules for future implementers. We found that generally the EIR had high acceptability among users; however, there were numerous barriers to using the system, even under ideal conditions, which characterized the gap between the system and the reality of the users’ workflows and environment. Implementers should consider workflows during the design and implementation phases and ensure they are evaluating how workflows may vary by site and user, to better align the system with reality. HCD and human-factors research can assist during a digital intervention’s pilot stages in time to make system changes, in addition to being used after scale-up to ensure interventions are acceptable in all user settings.

## Appendix

Multimedia Appendix 1 Data collection tools.

Multimedia Appendix 2 Code dictionary.

Multimedia Appendix 3 Summary of codes.

Multimedia Appendix 4 Intervention theory of electronic immunization record usability.

## Abbreviations

DHI: digital health intervention
EIR: electronic immunization record
HCD: human-centered design
HCW: health care worker
I-TECH: International Training and Education Center for Health
MOH: Ministry of Health
OJT: on-the-job training
POC: point of care
SCHRIO: subcounty health records information officer

## Data Availability

The data sets generated during and analyzed during this study are available from the corresponding author on reasonable request.

## References

[1] (2019). Global and immunization profile: African region. World Health Organization.

[2] Andre FE, Booy R, Bock HL, Clemens J, Datta SK, John TJ, Lee BW, Lolekha S, Peltola H, Ruff TA, Santosham M, Schmitt HJ (2008). Vaccination greatly reduces disease, disability, death and inequity worldwide. Bull World Health Organ.

[3] Rémy V, Zöllner Y, Heckmann U (2015). Vaccination: the cornerstone of an efficient healthcare system. J Mark Access Health Policy.

[4] Scobie HM, Edelstein M, Nicol E, Morice A, Rahimi N, MacDonald NE, Danovaro-Holliday CM, Jawad J, SAGE Working Group on Immunization and Surveillance Data Quality and Use (2020). Improving the quality and use of immunization and surveillance data: summary report of the Working Group of the Strategic Advisory Group of Experts on Immunization. Vaccine.

[5] McCool J, Dobson R, Whittaker R, Paton C (2022). Mobile health (mHealth) in low- and middle-income countries. Annu Rev Public Health.

[6] (2019). WHO guideline: recommendations on digital interventions for health system strengthening. World Health Organization.

[7] (2011). mHealth: new horizons for health through mobile technologies: second global survey on eHealth. WHO Global Observatory for eHealth.

[8] (2017). Electronic immunization registry: practical considerations for planning, development, implementation and evaluation. Pan American Health Organization.

[9] Grevendonk J, Taliesin B, Brigden D (2013). Planning an information systems project: a toolkit for public health managers. World Health Organization.

[10] Holeman I, Kane D (2019). Human-centered design for global health equity. Inf Technol Dev.

[11] Shaw J, Agarwal P, Desveaux L, Palma DC, Stamenova V, Jamieson T, Yang R, Bhatia RS, Bhattacharyya O (2018). Beyond "implementation": digital health innovation and service design. NPJ Digit Med.

[12] Fabricant R (2014). When will design get serious about impact?. Stanford Social Innovation Review.

[13] Lyon AR, Munson SA, Renn BN, Atkins DC, Pullmann MD, Friedman E, Areán PA (2019). Use of human-centered design to improve implementation of evidence-based psychotherapies in low-resource communities: protocol for studies applying a framework to assess usability. JMIR Res Protoc.

[14] Bazzano AN, Martin J, Hicks E, Faughnan M, Murphy L (2017). Human-centred design in global health: a scoping review of applications and contexts. PLoS One.

[15] Dolan SB, Wittenauer R, Njoroge A, Onyango P, Owiso G, Shearer JC, Lober WB, Liu S, Puttkammer N, Rabinowitz P (2022). Time utilization among immunization clinics using an electronic immunization registry: a time and motion study of modified user workflows. JMIR Form Res (forthcoming).

[16] Wittenauer RA (2022). Usability and acceptability of electronic immunization registry (EIR) data-entry workflows from the healthcare worker perspective in Siaya, Kenya. JMIR Form Res (forthcoming).

[17] Pawson R, Greenhalgh T, Harvey G, Walshe K (2005). Realist review--a new method of systematic review designed for complex policy interventions. J Health Serv Res Policy.

[18] Robert E, Samb OM, Marchal B, Ridde V (2017). Building a middle-range theory of free public healthcare seeking in sub-Saharan Africa: a realist review. Health Policy Plan.

[19] Pawson R (2000). Middle-range realism. Arch Eur Sociol.

[20] Wong G, Westhorp G, Manzano A, Greenhalgh J, Jagosh J, Greenhalgh T (2016). RAMESES II reporting standards for realist evaluations. BMC Med.

[21] Tong A, Sainsbury P, Craig J (2007). Consolidated criteria for reporting qualitative research (COREQ): a 32-item checklist for interviews and focus groups. Int J Qual Health Care.

[22] (2019). 2019 Kenya Population and Housing Census: Volume I Population by County and Sub-County. Kenya National Bureau of Statistics.

[23] Kenya National Bureau of Statistics, Ministry of Health, Kenya, National AIDS Control Council, Kenya Medical Research Institute, National Council for Population and Development, ICF International (2015). Kenya Demographic and Health Survey 2014. Kenya National Bureau of Statistics.

[24] (2011). Systems and software engineering — Systems and software Quality Requirements and Evaluation (SQuaRE) — System and software quality models. International Organization for Standardization.

[25] Ammenwerth E, Iller C, Mahler C (2006). IT-adoption and the interaction of task, technology and individuals: a fit framework and a case study. BMC Med Inform Decis Mak.

[26] Smith MJ, Sainfort PC (1989). A balance theory of job design for stress reduction. Int J Ind Ergon.

[27] Francis JJ, Johnston M, Robertson C, Glidewell L, Entwistle V, Eccles MP, Grimshaw JM (2010). What is an adequate sample size? Operationalising data saturation for theory-based interview studies. Psychol Health.

[28] Carayon P, Wetterneck T, Hundt AS, Ozkaynak M, DeSilvey J, Ludwig B, Ram P, Rough SS (2007). Evaluation of nurse interaction with bar code medication administration technology in the work environment. J Patient Saf.

[29] Carayon P, Wetterneck TB, Hundt AS, Ozkaynak M, Ram P, DeSilvey J, Hicks B, Robert TL, Enloe M, Sheth R, Sobande S, Henriksen K, Battles JB, Marks ES, Lewin DI (2005). Observing nurse interaction with infusion pump technologies. Advances in Patient Safety: From Research to Implementation (Volume 2: Concepts and Methodology).

[30] Carayon P, Wetterneck TB, Hundt AS, Ozkaynak M, Ram P, DeSilvey J, Hicks B, Robert TL, Enloe M, Sheth R, Sobande F, Khalid HM, Helander MG, Yeo AW (2004). Assessing nurse interaction with medication administration technologies: the development of observation methodologies. Work with Computing Systems.

[31] Miller K, Capan M, Weldon D, Noaiseh Y, Kowalski R, Kraft R, Schwartz S, Weintraub WS, Arnold R (2018). The design of decisions: matching clinical decision support recommendations to Nielsen's design heuristics. Int J Med Inform.

[32] Ryan RM, Deci EL (2000). Intrinsic and extrinsic motivations: classic definitions and new directions. Contemp Educ Psychol.

[33] Van Wingerden J, Van der Stoep J (2018). The motivational potential of meaningful work: relationships with strengths use, work engagement, and performance. PLoS One.

[34] Flott K, Callahan R, Darzi A, Mayer E (2016). A patient-centered framework for evaluating digital maturity of health services: a systematic review. J Med Internet Res.

[35] Bloland P, MacNeil A (2019). Defining and assessing the quality, usability, and utilization of immunization data. BMC Public Health.

[36] (2019). A realist review of what works to improve data use for immunization: evidence from low- and middle- income countries. Immunization Data: Evidence for Action, Pan American Health Organization.

[37] Mennito SH, Darden PM (2010). Impact of practice policies on pediatric immunization rates. J Pediatr.

[38] Heeks R (2006). Health information systems: failure, success and improvisation. Int J Med Inform.

[39] (2020). What are Global Goods. Digital Square.

[40] Ahlbrandt J, Henrich M, Hartmann BA, Bundschuh BB, Schwarz J, Klasen J, Röhrig R (2012). Small cause - big effect: improvement in interface design results in improved data quality - a multicenter crossover study. Stud Health Technol Inform.

[41] Steen M (2011). Tensions in human-centred design. CoDesign.

[42] Carayon P (2006). Human factors of complex sociotechnical systems. Appl Ergon.

[43] Black AD, Car J, Pagliari C, Anandan C, Cresswell K, Bokun T, McKinstry B, Procter R, Majeed A, Sheikh A (2011). The impact of eHealth on the quality and safety of health care: a systematic overview. PLoS Med.

[44] Buntin MB, Burke MF, Hoaglin MC, Blumenthal D (2011). The benefits of health information technology: a review of the recent literature shows predominantly positive results. Health Aff (Millwood).

[45] Sandhu JS, Hosang RN, Madsen KA (2015). Solutions that stick: activating cross-disciplinary collaboration in a graduate-level public health innovations course at the University of California, Berkeley. Am J Public Health.

[46] Beres LK, Simbeza S, Holmes CB, Mwamba C, Mukamba N, Sharma A, Munamunungu V, Mwachande M, Sikombe K, Bolton Moore C, Mody A, Koyuncu A, Christopoulos K, Jere L, Pry J, Ehrenkranz PD, Budden A, Geng E, Sikazwe I (2019). Human-centered design lessons for implementation science: improving the implementation of a patient-centered care intervention. J Acquir Immune Defic Syndr.

[47] Vechakul J, Shrimali BP, Sandhu JS (2015). Human-centered design as an approach for place-based innovation in public health: a case study from Oakland, California. Matern Child Health J.

[48] Battles JB, Keyes MA (2002). Technology and patient safety: a two-edged sword. Biomed Instrum Technol.

