# Supplementary material for: Integration of a Digital Health Intervention Into Immunization Clinic Workflows in Kenya: Qualitative, Realist Evaluation of Technology Usability
Source: JMIR Form Res. 2023 Mar 14;7:e39775. doi: 10.2196/39775 (PMC10131705; doi:10.2196/39775)
Supplement: Multimedia Appendix 1 [file formative_v7i1e39775_app1.docx]

# **Data Collection Tools**

**VACCINE ADMINISTRATION WORKFLOW OBSERVATIONS AND USER INTERVIEW**

***Complete 1 packet per facility.***

| Data Collection Information  *PLEASE FILL OUT THIS SECTION BEFORE BEGINNING OBSERVATIONS.* | |
| --- | --- |
| 1. **Date** | Day       Month            Year |
| 1. **Data collector’s name** |  |
| 1. **Facility MFL Code** | \|__\|__\|__\|__\|__\| |
| 1. **Facility Name** |  |
| 1. **Evaluation arm**    1. **Baseline**    2. **Modification 1**    3. **Modification 2**    4. **Modification 3** | \|__\| |

**Time began observations at facility**: ___________

**Part A. Health Care Worker Information and Consent**

***Before beginning observations, please collect the following information for all health care workers administering vaccinations, using KIP, or completing paper tools in the immunization clinic***

*INTRODUCE YOURSELF TO HEALTH CARE WORKER:* Thank you for agreeing to participate in this clinic observation. My name is ______. Today, I’d like to observe how you provide immunization services to children and use KIP on the tablet. We will document the task you are performing and where you are performing it. **We are not assessing your work**, we just want to better understand your work and challenges you encounter when using KIP. If you could please do your work today as you usually would. We will not speak and do our best to stay out of your way so as not to be disruptive. Do you consent to being observed by us today?

***[Provide healthcare worker with consent form and have them sign it] If they consent, ask them the following questions.***

| **Health Care Worker Information** | | |
| --- | --- | --- |
|  | **HCW 1** | **HCW 2** |
| 1. **Health care worker type** | 󠄀 Nurse  󠄀 Nurse in-charge  󠄀 MCH in-charge  󠄀 Clinical Officer  󠄀 Other | 󠄀 Nurse  󠄀 Nurse in-charge  󠄀 MCH in-charge  󠄀 Clinical Officer  󠄀 Other |
| 1. **Length of time at this facility** | 󠄀 <1 year  󠄀 1-5 years  󠄀 6-10 years  󠄀 More than 10 years | 󠄀 <1 year  󠄀 1-5 years  󠄀 6-10 years  󠄀 More than 10 years |
| 1. **Length of time using KIP** | 󠄀 <1 month  󠄀 1-3 months  󠄀 More than 3 months | 󠄀 <1 month  󠄀 1-3 months  󠄀 More than 3 months |
| 1. **Did health care worker sign consent form to participate?** | 󠄀 Yes. Proceed with data collection.  󠄀 No. STOP. | 󠄀 Yes. Proceed with data collection.  󠄀 No. STOP. |

**Part B: Workflow Observations**

***Instructions for workflow observation***

- - Observe 5 caregiver-baby pairs or all pairs presenting at the clinic from the time they approach the healthcare worker for vaccination to the time they complete the vaccination encounter.
  - Use a stopwatch to record the time it takes to complete each vaccination session and consultation per child.
  - Document any changes in the facility’s physical environment during the observation period.
  - As you time each child’s session, observe how KIP is used and document any surprises that impact data entry or data use; if there are any interruptions to the workflow, document these.

**Key Points**

- - It is important to map what is ***actually*** happening, not what you think or want to happen
  - Identify every step of the activity and the order of activities.
  - Workflows may overlap or depend on the execution of another activity or process; consider these dependencies and make note of them.
  - Document if some activities are completed simultaneously.

**Activities to Map (indicate if any of these activities do not occur)**

- - Searching for a child’s record
  - Registering a child
  - Growth monitoring
  - Identifying vaccines due
  - Administering vaccines
  - Recording vaccines administered
  - Consulting with a child’s parent/caregiver
  - Nutrition counseling
  - Deworming
  - Administering Vitamin A
  - Distributing ITN
  - Other
- If you have any additional comments, kindly write them in the spaces provide.

**Child #1**

*Record the amount of time it takes each child to go through the vaccination process and the order of activities. Please comment on anything observed during the process that would impact data entry, data use, or data quality.*

**Vaccination Session- Start time**:________ **End time:____**

**Child has a MCB**: __ Yes __ No **Child visiting facility for 1^st^ time**: __ Yes __ No **# of vaccines admin**:____

| **Time** | **Activity** | **Tool** | **Order** | **Observations on Usability** | **Interruptions (who/why)** |
| --- | --- | --- | --- | --- | --- |
| **Start time: _____**  **Stop time: _____** | Search for child record | Paper |  |  |  |
|  |  | KIP |  |  |  |
|  | Registration | Paper |  |  |  |
|  |  | KIP |  |  |  |
| **Start time: _____**  **Stop time: _____** | Growth monitoring | Paper |  |  |  |
|  |  | KIP |  |  |  |
|  | Identifying vaccines due |  |  |  |  |
|  | Administering vaccines |  |  |  |  |
|  | Recording vaccines administered | Paper |  |  |  |
|  |  | KIP |  |  |  |
|  | Administering Vitamin A | Paper |  |  |  |
|  |  | KIP |  |  |  |
| **Start time: _____**  **Stop time: _____** | Consult with caregiver |  |  |  |  |

###

***Instructions for facility-level information***

- Document the type of workflow, environmental conditions, and connectivity

| **Facility-Level Information** | |
| --- | --- |
| **Type of clinic workflow**  **(check one)** | 󠄀 Single child vaccinated at a time  󠄀 Multiple children vaccinated at one-time, “batched” vaccination  󠄀 Other (please explain):  ___ |
| **Type of data entry workflow with KIP**  **(check one)** | 󠄀 Point-of-care  󠄀 Other (please explain): |
| **Number of lanes used during the immunization session and the number of healthcare workers in each lane**  **(check one)** | 󠄀 1 lane, number of healthcare workers: ___  󠄀 Other (please explain): |
| **Physical environment**  **(check all that apply)** | 󠄀 neat, organized (vs. messy)  󠄀 uncrowded (vs. crowded)  󠄀 quiet (vs. noisy)  󠄀 well lit (easy to see) (vs. dim) |
| **Connectivity**  **(check all that apply)** | 󠄀 KIP and tablet working  󠄀 KIP fully charged  󠄀 KIP connected to internet  󠄀 KIP synching with server |
| **Other observations of the clinic area** | Comments: |

*After all caregiver-child observations are completed, take 10 minutes to think about what you saw and sketch out the general workflow on the clinic (you will need this for the interviews).*

**GENERAL NOTES/REFLECTIONS, especially regarding bottlenecks or inefficiencies observed that would impact data entry and data use:**

**FACILITY WORKFLOW, draw the order of activities and whether KIP or paper tools were used to perform each activity:**

### **Key Informant Interview Guide**

**USER SATISFACTION AND ACCEPTABILITY ASSESSMENT TOOL**

| Data Collection Information  *PLEASE FILL OUT THIS SECTION BEFORE BEGINNING OBSERVATIONS.* | |
| --- | --- |
| 1. **Date** | Day       Month            Year |
| 1. **Data collector’s name** |  |
| 1. **Facility MFL Code** |  |
| 1. **Facility Name** |  |
| 1. **Evaluation arm**    1. **Baseline**    2. **Modification 1**    3. **Modification 2**    4. **Modification 3** |  |
| 1. **User Unique ID Number** |  |

***Instructions***:

**Step 1.** Find a quiet place to sit down with the HCW. Explain to the HCW that you will discuss what you just observed during the clinic session and that you would like their feedback on what challenges they encountered when using KIP and how KIP could be improved. Explain to the HCW that this interview will take approximately 30-45 minutes and that all of their responses will be recorded, but remain anonymous, and will not be used to assess their job performance.

**Step 2.** (Workflow Validation) Show the HCW your drawing of the clinic workflow and explain each task you observed, in the order completed. Describe each task in detail and confirm with the HCW that what you observed was correct. Make any modifications needed to your notes.

**Step 3.** (Task Oriented Evaluation) Review the workflow again, this time using the table below to discuss how demanding each task was for the HCW to complete using KIP. For each task listed below, ask the HCW to assign a score between 1-10 (1 being low and 10 being high); use the task load definitions below and circle a number. Also, be sure to probe about challenges encountered during each task that you observed and document comments from the HCW during this discussion.

| **Overall Vaccination Session- Using KIP** |  |
| --- | --- |
| Mental Demand | (low) 1---2---3---4---5---6---7--- 8---9---10 (high) Mental Demand |
| Time Pressure | (low) 1---2---3---4---5---6---7--- 8---9---10 (high) Pressure |
| Ability to use KIP | (low) 1---2---3---4---5---6---7--- 8---9---10 Performed well |
| Effort | (low) 1---2---3---4---5---6---7--- 8---9---10 (high) Effort |
| Frustration | (low) 1---2---3---4---5---6---7--- 8---9---10 Very Frustrated |
| Challenges encountered: |  |
| Comments: |  |

**Task Load Definitions**

- **Mental demand-** How much mental activity was required (e.g. thinking, deciding, calculating, remembering, looking, searching, etc.)? Was the task mentally easy or demanding, simple or complex, exacting or forgiving?
- **Time pressure**- how much time pressure did you feel due to the rate of pace at which the tasks or task elements occurred? Was the pace slow and leisurely or rapid and frantic?
- **Performance**- How successful do you think you were in accomplishing the goals of the task? How satisfied were you with your performance in accomplishing these goals?
- **Effort**- How hard did you have to work (mentally and physically) to accomplish your level of performance?
- **Frustration**- How insecure, discouraged, irritated, stressed, and annoyed versus secure, gratified, content, relaxed, and complacent did you feel during the task?

**Step 4**. (Heuristic Evaluation) Review the workflow with the HCW again, this time discussing the major challenges and barriers you observed. Use the following questions to guide the discussion, asking the HCW to clarify any challenges mentioned.

**Usability Heuristics**

- 1. **Visibility of the system status**- the system should always keep users informed about what is going on, through appropriate feedback within reasonable time.
  2. **Match between the system and the real world**- the system should speak the users’ language, follow real-world conventions, making information appear in a natural and logical order.
  3. **User control and freedom**- support for undo and redo of actions.
  4. **Consistency and standards**- follow platform conventions, use of consistent words, situations, and actions.
  5. **Flexibility and efficiency of use**- allow users to tailor frequent actions.
  6. **Aesthetic and minimalist design**- no information that is irrelevant or rarely needed.
  7. **Error prevention**- eliminate error-prone conditions or check for them and present users with a confirmation option before they commit to an action.
  8. **Recognition rather than recall**- minimize the users’ memory load by making objects, actions, and options visible. The user should not have to remember information. Instructions for use should be visible or easily retrievable.
  9. **Help and documentation**- easy to search for information, focused on the user’s task, listing concrete steps to carry out.
  10. **Help users recognize, diagnose, and recover from errors**- error messages expressed in plain language, precisely indicate the problem, and constructively suggest a solution.
  11. **Integration into real-time workflow**- considers the needs of the user in the clinical space; information rapidly accessible and understood from within the clinician’s workflow.

**Usability Questions**

Ask the healthcare worker the following questions. Use KIP on the tablet to demonstrate concepts as indicated by the brackets when necessary, follow the guidance as described. For the open-ended questions take notes on the HCW’s answers and, for the close-ended questions, read each of the statements to the healthcare worker and ask how much they agree with each statement. Check the appropriate box.

| **1** | **Visibility of the system status**  [Ask the HCW to search for a child using advanced search]  When you are using KIP, you feel the system provides enough feedback/messages to you as it processes information, you understand what the system is doing.  ☐Strongly disagree   ☐disagree  ☐Neither agree nor disagree   ☐Agree   ☐Strongly agree  Explain: |
| --- | --- |
| **2** | **Match between the system and the real world**  [Ask the HCW to go to a child’s record and look at the growth monitoring and immunization information]  You feel that KIP captures the correct information during a nutrition and immunization session.  ☐Strongly disagree   ☐disagree  ☐Neither agree nor disagree   ☐Agree   ☐Strongly agree  Explain: |
| **3** | **User control and freedom/Error Prevention**  [Ask the HCW to enter child information, save it, and then undo what she/he entered]  You feel you have the ability to undo and redo actions when entering child information.  ☐Strongly disagree   ☐disagree  ☐Neither agree nor disagree   ☐Agree   ☐Strongly agree  Explain:  If you make an error when using KIP, you feel it is easy to correct the mistake and that the system helps you prevent making mistakes.  ☐Strongly disagree   ☐disagree  ☐Neither agree nor disagree   ☐Agree   ☐Strongly agree  Explain: |
|  | It is easy to move from one screen page/menu to the next  ☐Strongly disagree   ☐disagree  ☐Neither agree nor disagree   ☐Agree   ☐Strongly agree |
| **3.a** | You can easily update/edit all the child registration details  ☐Strongly disagree   ☐disagree  ☐Neither agree nor disagree   ☐Agree   ☐Strongly agree |
| **5** | **Aesthetic and minimalist design**  You feel there is not too much information in KIP, and that all of the information is needed.  ☐Strongly disagree   ☐disagree  ☐Neither agree nor disagree   ☐Agree   ☐Strongly agree  Explain: |
| **6** | **Flexibility and efficiency of use**  You feel the system is flexible enough to allow you to complete frequent actions easily.  ☐Strongly disagree   ☐disagree  ☐Neither agree nor disagree   ☐Agree   ☐Strongly agree  Explain: |
| **6.a** | I find KIP easy to use.  ☐Strongly disagree   ☐Disagree ☐Neither agree nor disagree   ☐Agree ☐Strongly agree |
| **8** | **Help and documentation**  You feel it is easy to find help when you need it.  ☐Strongly disagree   ☐disagree  ☐Neither agree nor disagree   ☐Agree   ☐Strongly agree  Explain: |
| **9** | **Help users recognize, diagnose, and recover from errors**  You feel that the error messages KIP generates help to indicate a problem and suggest how to solve it.  ☐Strongly disagree   ☐disagree  ☐Neither agree nor disagree   ☐Agree   ☐Strongly agree  Explain: |
| **10** | **Integration into real-time workflow**  How has your job changed since this facility started using KIP for immunization records? Why or why not?  In comparing your workflow with only paper records or with KIP, could you describe which one you prefer and why? Which system do you trust more, the paper-based tools or KIP? Why? Which system do you believe has the best quality information? |
| **10.a** | Do you feel the data collected by KIP are meaningful? Do you feel the data collected by KIP are suitable for identifying vaccinations needed and to understand vaccination coverage for your facility? Do you feel the data collected in KIP are useful? |
| **10.b** | KIP provides the information I need to easily vaccinate children.  ☐Strongly disagree   ☐Disagree ☐Neither agree nor disagree   ☐Agree ☐Strongly agree |
| **10.c** | I have enough time to vaccinate all patients attending an immunization clinic.  ☐Strongly disagree   ☐Disagree ☐Neither agree nor disagree   ☐Agree ☐Strongly agree |
| **10.d** | The clinic workflow is good when using KIP and paper tools at the point-of-care.  ☐Strongly disagree   ☐Disagree ☐Neither agree nor disagree   ☐Agree ☐Strongly agree |
| **10.e** | We have enough tablets for our clinic to use KIP.  ☐Strongly disagree   ☐Disagree ☐Neither agree nor disagree   ☐Agree ☐Strongly agree |
| **10.f** | We have enough staff to adequately use KIP during our immunization clinic.  ☐Strongly disagree   ☐Disagree ☐Neither agree nor disagree   ☐Agree ☐Strongly agree |
| **10.g** | The system was functioning well most of the time when you needed to use it.  ☐Strongly disagree   ☐disagree ☐Neither agree nor disagree   ☐Agree ☐Strongly agree |
| **10.h** | System downtime was minimal.  ☐Strongly disagree   ☐disagree ☐Neither agree nor disagree   ☐Agree ☐Strongly agree |
| **10.i** | I trust that the data in the EIR is stored securely and will not be lost.  ☐Strongly disagree   ☐disagree ☐Neither agree nor disagree   ☐Agree ☐Strongly agree |
| **10.j** | I trust the data in KIP are of good quality.  ☐Strongly disagree   ☐disagree ☐Neither agree not disagree   ☐Agree ☐Strongly agree |
| **11** | **Satisfaction with KIP**  Are you satisfied with KIP as it is used in your facility? Why or why not? |
| **11.a** | I would recommend the system for use by other users/health facilities  ☐Strongly disagree   ☐disagree ☐Neither agree not disagree   ☐Agree ☐Strongly agree |
| **11.b** | Overall, I am satisfied with KIP.  ☐Strongly disagree   ☐Disagree ☐Neither agree nor disagree   ☐Agree ☐Strongly agree |
| **11.c** | KIP improves the quality of patient care.  ☐Strongly disagree   ☐Disagree ☐Neither agree nor disagree   ☐Agree ☐Strongly agree |
| **11.d** | I feel I received adequate training on how to use KIP appropriately for my clinic.  ☐Strongly disagree   ☐Disagree ☐Neither agree nor disagree   ☐Agree ☐Strongly agree |
| **11.e** | I know where to find KIP user guides and help functions.  ☐Strongly disagree   ☐Disagree ☐Neither agree nor disagree   ☐Agree ☐Strongly agree |
| **11.f** | I feel I receive adequate supervisory support for using KIP in my clinic.  ☐Strongly disagree   ☐Disagree ☐Neither agree nor disagree   ☐Agree ☐Strongly agree |
| **11.g** | What recommendations do you have for improving the system? |
| **11.h** | Please provide any other comments on how you feel about KIP: |

**Time finished observations at facility**: ______
