# Supplementary figures and images for: Integration of a Digital Health Intervention Into Immunization Clinic Workflows in Kenya: Qualitative, Realist Evaluation of Technology Usability
Source: JMIR Form Res. 2023 Mar 14;7:e39775. doi: 10.2196/39775 (PMC10131705; doi:10.2196/39775)

**Table A. Code Dictionary**

**
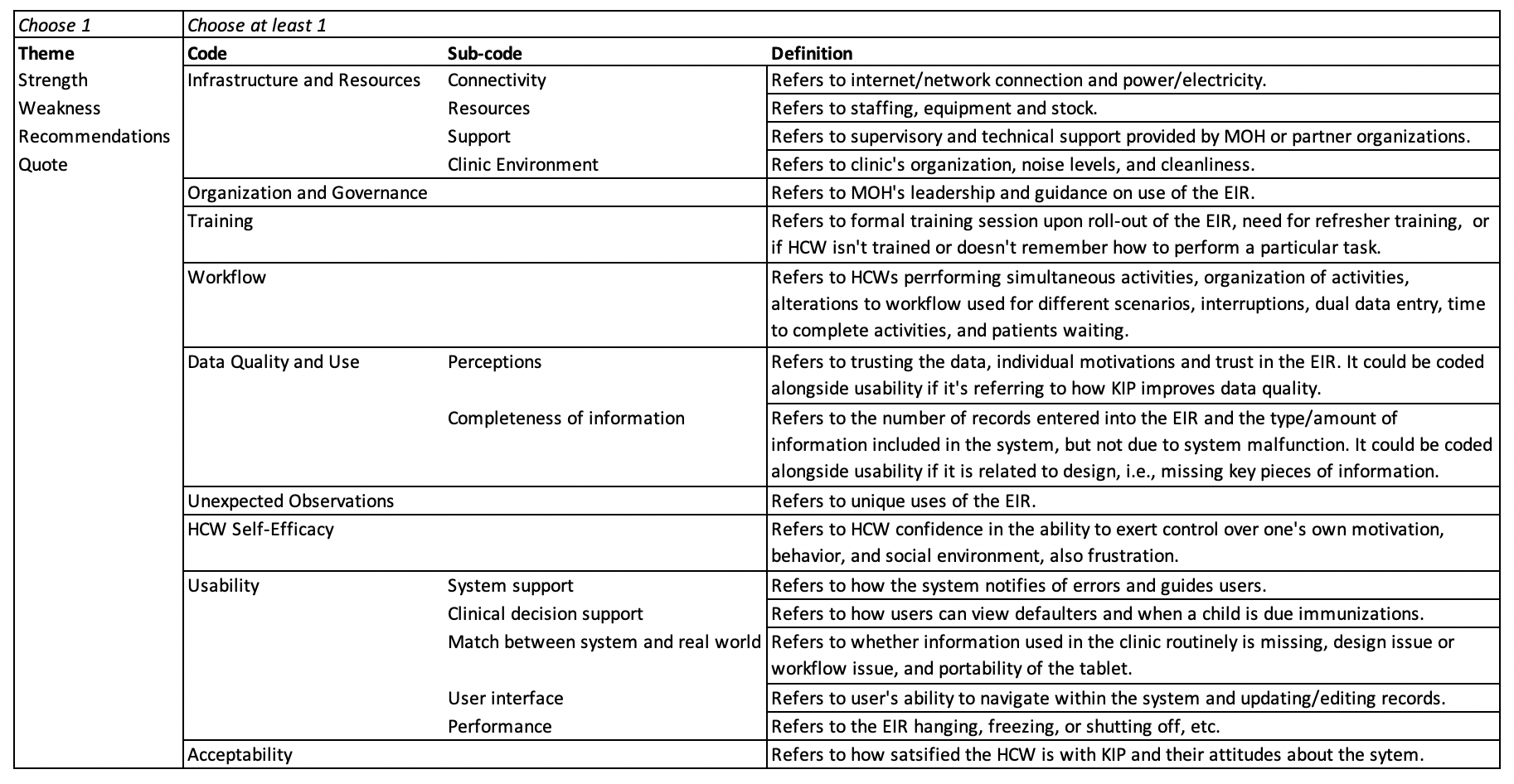
**

Supplement: Multimedia Appendix 2 [file formative_v7i1e39775_app2.docx]

**Table B. Summary of Codes**

**
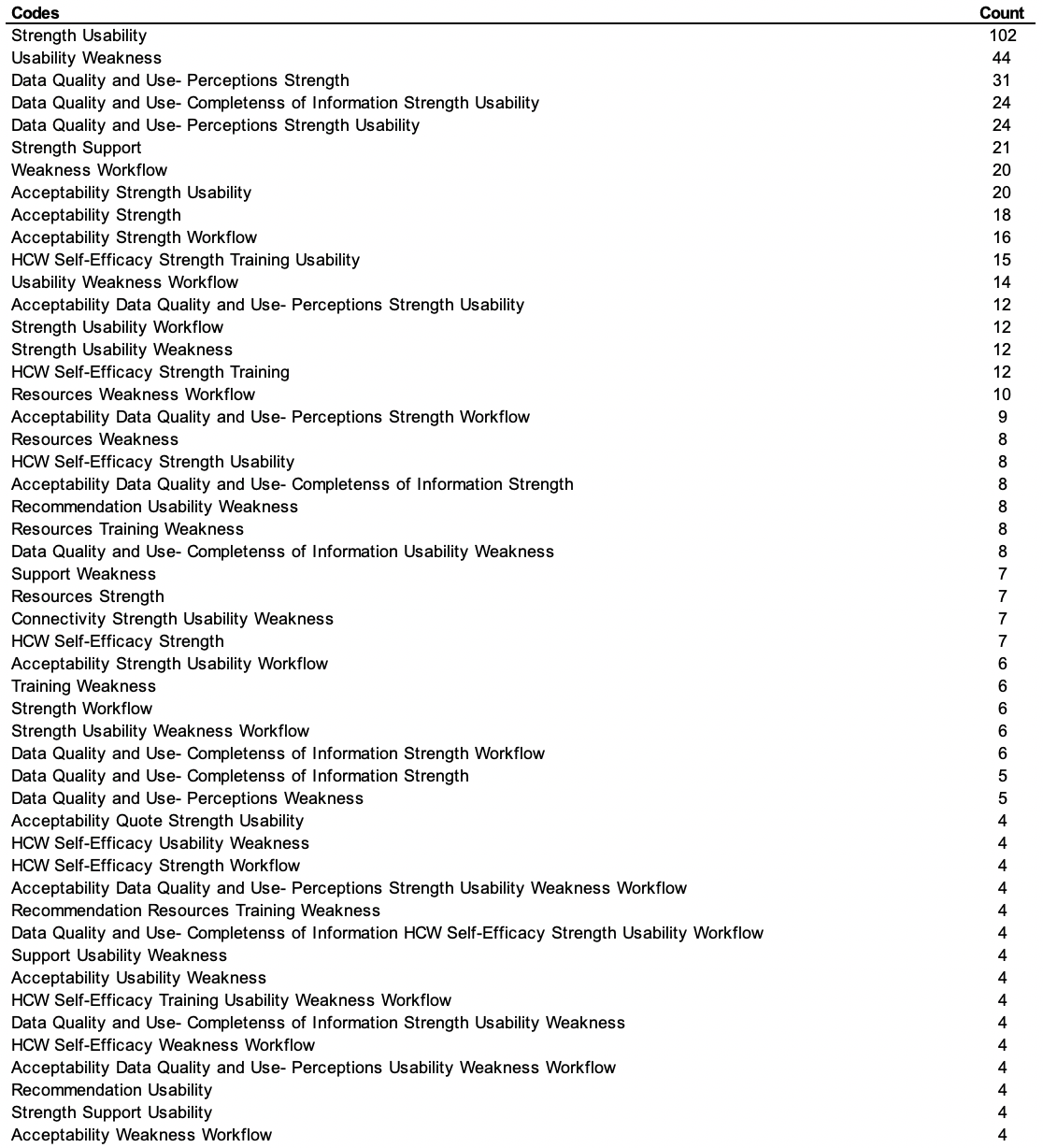
**

**
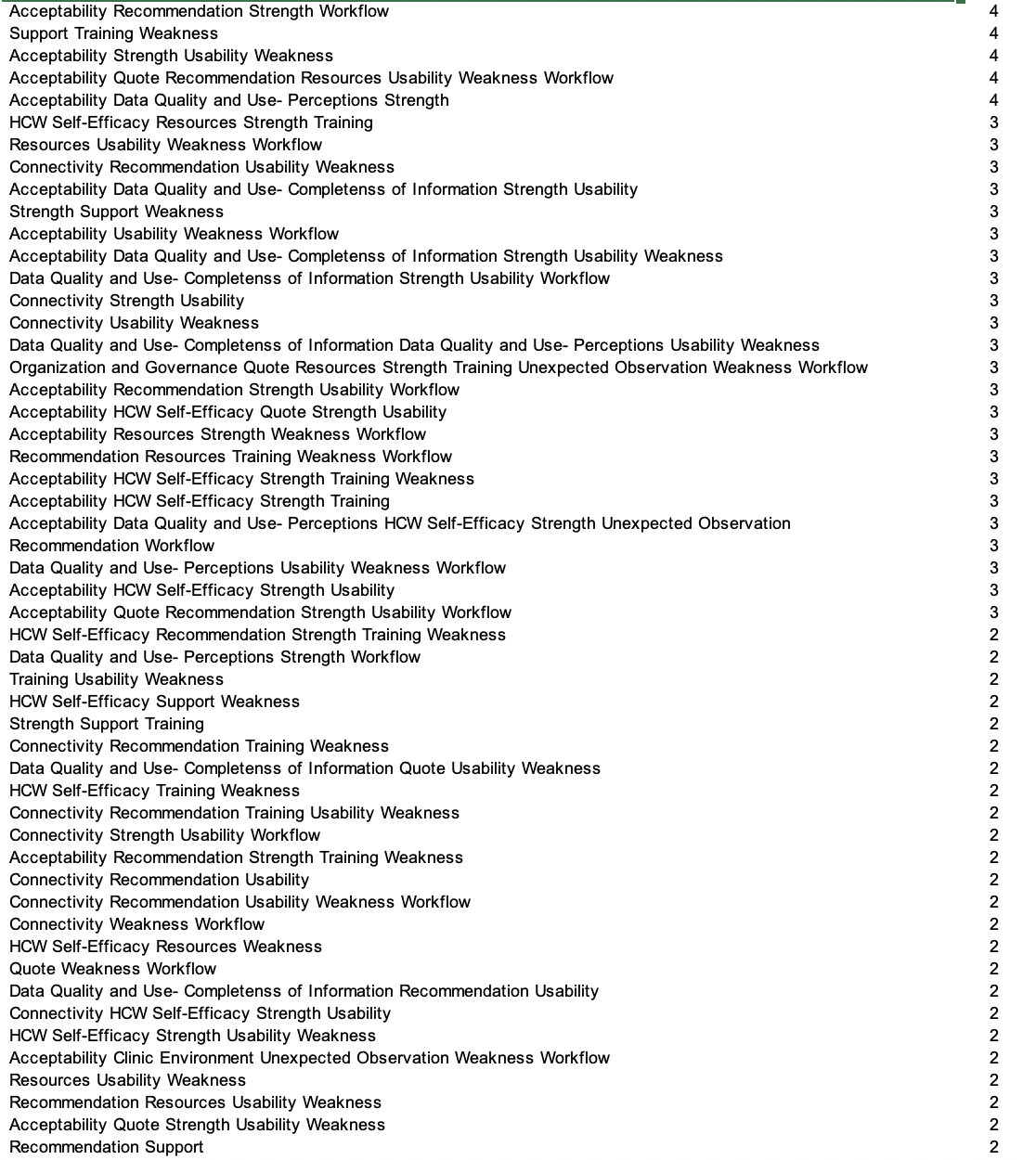
**

**
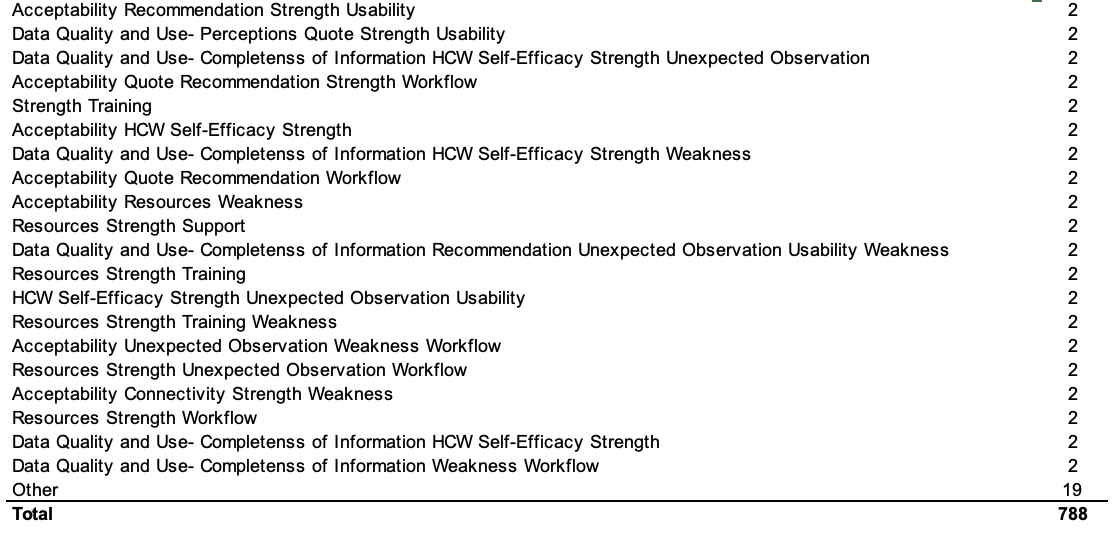
**

Supplement: Multimedia Appendix 3 [file formative_v7i1e39775_app3.docx]
