# Supplementary material for: Integration of a Digital Health Intervention Into Immunization Clinic Workflows in Kenya: Qualitative, Realist Evaluation of Technology Usability
Source: JMIR Form Res. 2023 Mar 14;7:e39775. doi: 10.2196/39775 (PMC10131705; doi:10.2196/39775)
Supplement: Multimedia Appendix 4 [file formative_v7i1e39775_app4.docx]

**Figure A. Intervention Theory of EIR-Usability**

**
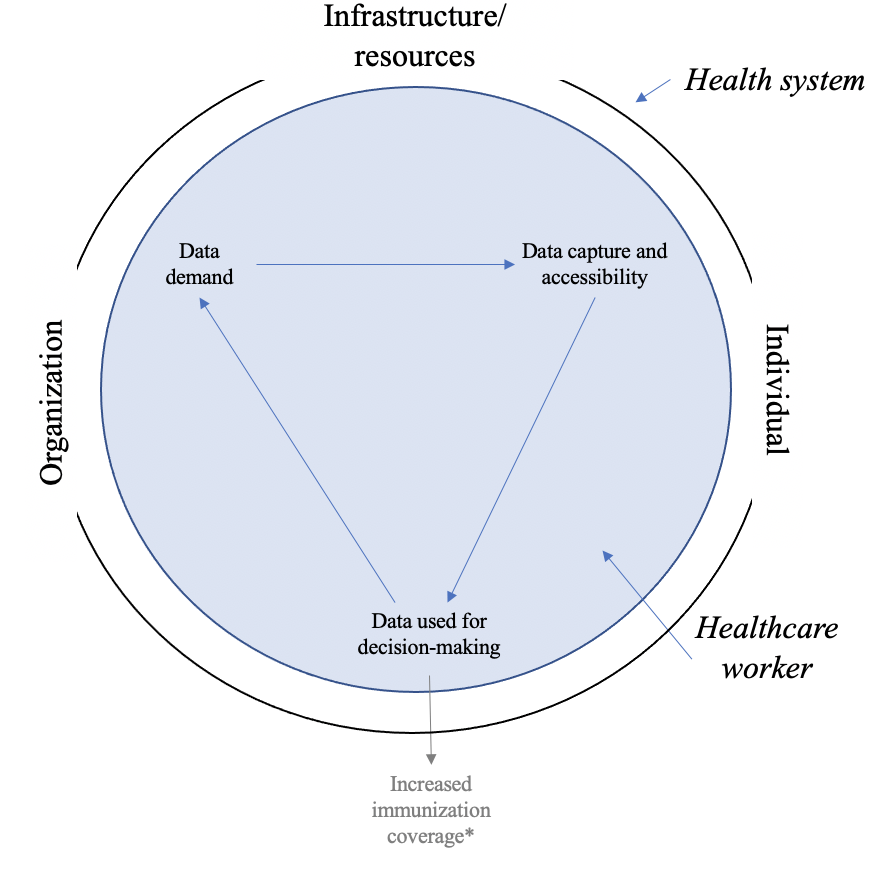
**

*Immunization coverage was not part of our realist evaluation
